# Diagnostic value of retinol-binding protein 4 in diabetic nephropathy: a systematic review and meta-analysis

**DOI:** 10.3389/fendo.2024.1356131

**Published:** 2024-04-22

**Authors:** Xiaodan Cao, Guanghui Zhong, Tinglong Jin, Weijiao Hu, Jin Wang, Bo Shi, Renxiong Wei

**Affiliations:** ^1^ Department of Clinical Laboratory, Ningbo Municipal Hospital of Traditional Chinese Medicine, Affiliated to Zhejiang Chinese Medical University, Ningbo, China; ^2^ Department of Nephrology, Ningbo Municipal Hospital of Traditional Chinese Medicine, Affiliated to Zhejiang Chinese Medical University, Ningbo, China; ^3^ Department of Endocrinology, Ningbo Municipal Hospital of Traditional Chinese Medicine, Affiliated to Zhejiang Chinese Medical University, Ningbo, China

**Keywords:** retinol-binding protein 4, diabetic nephropathy, diagnosis, biomarkers, meta-analysis

## Abstract

**Objective:**

Diabetic nephropathy (DN) is a major microvascular complication of diabetes and the leading cause of end-stage renal disease. Early detection and prevention of DN are important. Retinol-binding protein 4 (RBP4) has been considered as a single diagnostic marker for the detection of renal impairment. However, the results have been inconsistent. The present meta-analysis aimed to determine the diagnostic potential of RBP4 in patients in type 2 diabetes mellitus (T2DM) with DN.

**Methods:**

We searched PubMed, Web of Science, Embase, Wanfang and CNKI databases from inception until January 2024. The meta-analysis was performed by Stata version 15.0, and sensitivity, specificity, positive and negative likelihood ratios (PLR and NLR), diagnostic odds ratio (DOR) and area under the curve (AUC) were pooled. The Quality Assessment of Diagnostic Accuracy Studies-2 tool was utilized to assess the quality of each included study. In addition, heterogeneity and publication bias were evaluated.

**Results:**

Twenty-nine studies were included in the meta-analysis. The pooled sensitivity and specificity were 0.76 [95% confidence interval (CI), 0.71–0.80] and 0.81 (95% CI, 0.76–0.85), respectively. The results showed a pooled PLR of 4.06 (95% CI, 3.16–5.21), NLR of 0.29 (95% CI, 0.24–0.36) and DOR of 13.76 (95% CI, 9.29–20.37). The area under the summarized receiver operating characteristic curve was given a value of 0.85 (95% CI, 0.82–0.88). No obvious publication bias existed in the Deeks’ funnel plot asymmetry test.

**Conclusion:**

Our findings suggest that RBP4 has a promising diagnostic value with good sensitivity and specificity for patients with T2DM with DN.

## Introduction

Diabetic nephropathy (DN) is a leading cause of morbidity and mortality among patients with type 2 diabetes mellitus (T2DM). It is characterized by increased glomerular filtration rate (GFR) with intraglomerular hypertension and clinically progressive albuminuria, followed by eventual loss of renal function (1). Changes in GFR or albuminuria are currently considered hallmarks of onset or progression of DN. However, the levels of estimated GFR (eGFR) or urinary albumin are in the normal range in some patients with early stage DN, which suggests that eGFR or albuminuria is not a suitable marker for early diagnosis of DN. This has motivated researchers to consider potential novel diagnostic biomarkers (2).

Retinol-binding protein 4 (RBP4) is an adipokine that belongs to the lipocalin superfamily, binds specifically to vitamin A, transports small hydrophobic molecules and is generated mainly in the liver and mature fat cells (20%–40%) (3). Several studies have shown that RBP4 is closely associated with obesity in diabetic patients, insulin resistance (IR), renal impairment and cardiometabolic indices (4, 5). Previous research has indicated that RBP4 influences insulin-responsive glucose transporter-4 in adipocytes, which is related to insulin sensitivity (6, 7). Elevated serum RBP4 levels are high in patients with T2DM, IR and impaired glucose tolerance (8, 9). Serum RBP4 concentrations are also correlated with changes in eGFR and serum creatinine, demonstrating its correlation with renal function (10). As a result of the low molecular weight (21 kDa) of RBP4, it is freely filtered through the glomeruli and then almost entirely reabsorbed in the proximal tubules, making urinary RBP4 an effective marker of small changes in proximal tubule function (11, 12). RBP4 is present before the increase of other markers such as proteinuria and serum creatinine (4, 13). Most previous studies have revealed a positive relationship between RBP4 and renal dysfunction markers such as albuminuria (4, 14, 15). However, the results remain inconsistent (16). Thus, our meta-analysis aimed to assess the diagnostic value of RBP4 as a biomarker for early detection of DN in patients with T2DM.

## Materials and methods

### Literature search

Two independent reviewers (TJ and WH) searched PubMed, Web of Science, Embase, Wanfang and CNKI databases from inception until January 2024. The study type was not restricted. The terms of our search were as follows: (“Diabetic Nephropathy” OR “Diabetic Kidney Disease” OR “Diabetic Nephropathies” OR “Diabetes Mellitus” OR “Type 2 Diabetic” and “Nephropathy”, then combined these items using AND with “Retinol-binding protein 4” OR “RBP4” AND (“diagnosis” OR “classification” OR “discriminate”) AND (“accuracy” OR “sensitivity” OR “specificity” OR “area under the curve”). This meta-analysis followed the PRISMA statement of preferred reporting items for systematic evaluation and meta-analysis.

### Criteria for study inclusion and exclusion

The study inclusion criteria were as follows: (1) diagnostic study; (2) T2DM patients with or without DN; (3) availability of indexes containing true positive (TP), false positive (FP), false negative (FN) and true negative (TN) values; and (4) inclusion of diagnostic cut-off values for RBP4. Exclusion criteria were: (1) reviews, letters, conference abstracts or animal studies; (2) studies with duplicate data; and (3) failure to extract four-cell table data. XC and JW selected the studies independently according to the above criteria. If there were disagreements among the reviewers, a joint consultation was held with a third reviewer (BS) for verification.

### Literature quality assessment

Two independent researchers (BS and RW) completed the quality assessment of included studies using the Quality Assessment of Diagnostic Accuracy Studies-2 (QUADAS-2). Items assessed contained two categories of risk of bias and applicability concerns. Patient selection, index test, reference standard, flow and timing were assessed for risk of bias, and the first three items were also assessed for applicability concerns. According to the answers to the landmark issues included in each section of yes, no or uncertain, the bias risk was judged as low, high or uncertain.

### Data extraction

Key variables from each study included: first author, publication year, country of origin sample source, number of participants, TP, FP, FN, TN, cut-off values and diagnostic criteria for DN. The sources of heterogeneity were discovered by meta-regression analysis with sample source (serum or urine), region (China or not), diagnostic criteria [albumin/creatinine ratio (ACR) or others], bias risk for index test (bias or no bias), study design (cross-sectional or case–control study) and sample size (>200 or ≤200) as independent variables. Data extraction was accomplished independently by two investigators (XC and JW). Disagreements were discussed and resolved by consensus.

### Statistical analysis

Data from the selected studies were reconstructed in 2 × 2 tables (TP, FN, FP, TN), and their sensitivity and specificity were calculated. The diagnostic meta-analyses were conducted using Stata version 15.0 software, with pooled effect sizes containing specificity, sensitivity, positive likelihood ratio (PLR), negative likelihood ratio (NLR), diagnostic odds ratio (DOR) and area under the curve (AUC) with their 95% confidence intervals (CIs). The “MIDAS” module was used for synthesizing the data to explore the combined sensitivity and specificity and their 95% CI. The summary ROC (SROC) was used for calculating the AUC of the diagnostic value. Heterogeneity was evaluated statistically by the Cochran Q test and *I^2^* statistics. If *P* was <0.05 or *I^2^* >50%, the data were analyzed in a random-effects model. Otherwise, a fixed-effects model was used. The sources of heterogeneity were analyzed using meta-regression. Sensitivity analysis was conducted to assess the robustness of the meta-analysis. Fagan’s nomogram was performed to further estimate the diagnostic efficacy of RBP4. The publication bias was assessed using Deeks’ funnel plot asymmetry test, and *P*<0.05 was considered statistically significant.

## Results

### Characteristics of the included studies

**Figure 1:**
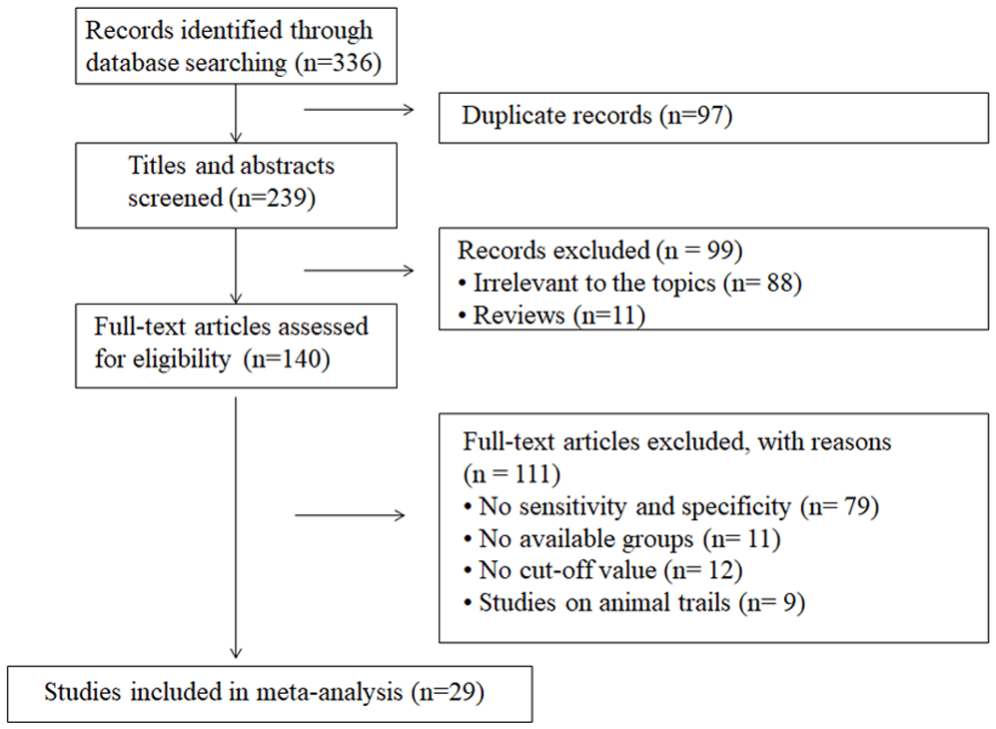
Flow diagram of literature selection.

The search strategy yielded 336 publications according to the eligibility criteria, among which 97 were duplicates. After screening title or abstracts, we excluded 99 because they were reviews or covered irrelevant topics. Of the 140 remaining articles, 111 were excluded after full-text evaluation, including 79 without sensitivity and specificity, 11 without available groups, 12 without cut-off values and nine animal studies. Finally, 29 articles were included, providing data on 2849 samples in the DN group and 2700 controls. The detailed screening process is shown in **Figure 1**. Among the 29 articles, two were published in English (4, 14) and the other 27 in Chinese (17–43). Serum or urine samples were collected from patients for RBP4 detection. The included patients were diagnosed with DN according to estimated glomerular filtration rate (eGFR), ACR and albumin excretion rate (AER) values. The main characteristics of the articles included in the meta-analysis are listed in **Table 1**.

**Table 1:** Characteristics of the included studies.

Author	Year	Region	Sample	Case	Control	TP	FP	FN	TN	Cut-off	Reference for GFR/ACR/AER
Zhao	2024	China	urine	130	97	106	25	24	72	0.7mg/L	ACR
Xu	2023	China	urine	89	98	62	34	27	64	58mg/L	ACR
Lin	2023	China	serum	50	50	39	1	11	49	39.62mg/L	ACR
Qiu	2022	China	urine	56	48	55	24	1	24	2.49mg/L	ACR
Wu	2022	China	serum	42	68	36	13	6	55	70mg/L	AER
Chang	2022	China	serum	199	657	103	100	96	557	50.5 mg/L	ACR
Yang	2022	China	serum	48	50	35	7	13	43	55.97 mg/L	AER
Zeng	2022	China	serum	87	60	63	12	24	48	58.42mg/L	ACR
Gao	2021	China	serum	242	87	142	19	100	68	50mg/L	AER
Tao	2021	China	serum	42	58	33	12	9	46	50mg/mL	AER
Xiang	2020	China	serum	63	65	45	13	18	52	53.88mg/L	ACR
Wang	2020	China	serum	90	90	74	12	16	78	54.28mg/L	ACR
Li	2020	China	urine	46	66	35	13	11	53	70mg/L	AER
Gao	2020	China	serum	99	102	45	20	54	82	45.95mg/L	ACR
Abbasi	2020	Iran	serum	89	44	75	17	14	27	46.1 ng/mg	GFR
Li	2019	China	serum	64	60	43	4	21	56	70mg/L	AER
Wang	2019	China	serum	165	81	120	24	45	57	70mg/L	GFR
Yang	2018	China	serum	127	41	89	10	38	31	70mg/L	AER
Lu	2018	China	urine	150	74	125	0	25	74	3.0mg/L	AER
Shen	2018	China	serum	370	370	223	73	147	297	70mg/L	NR
Chen	2018	China	serum	40	40	34	9	6	31	30.1mg/L	GFR
Kong	2017	China	urine	89	35	76	6	13	29	0.32mg/L	AER
Huang	2017	China	serum	101	47	87	5	14	42	64.2mg/L	ACR
Li	2016	China	urine	32	43	26	15	6	28	0.7mg/L	AER
Mahfouz	2016	Saudi Arabia	serum	100	50	84	5	16	45	24.5 ng/ml	ACR
Xie	2015	China	serum	38	44	24	8	14	36	57.9mg/L	AER
Li	2015	China	serum	95	60	77	3	18	57	40.95mmol/L	ACR
Qiu	2013	China	urine	60	60	30	27	30	33	1.5mg/L	AER
Zhang	2012	China	serum	46	55	41	18	5	37	51mg/L	AER

TP, true positive; FP, false positive; FN, false negative; TN, true negative; NR, not reported; GFR, glomerular filtration rate; ACR, albumin/creatinine ratio; AER, albumin excretion rate.

### QUADAS−2 scores

**Figure 2:**
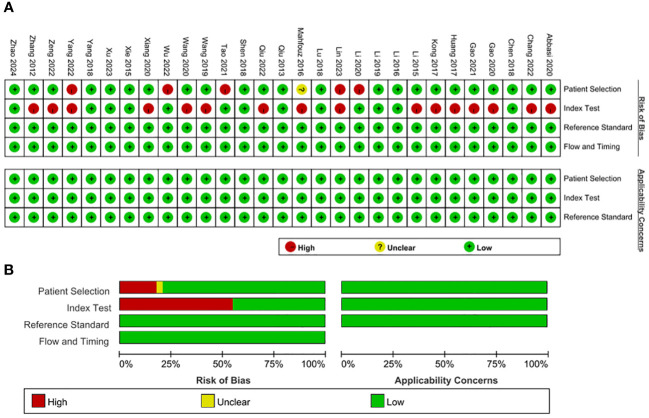
Bias risk assessment by Quality Assessment of Diagnostic Accuracy Studies-2 (QUADAS−2). **(A)** QUADAS−2 summary plot **(B)** QUADAS−2 bar plot.

The bias risk assessment of the included studies is described in **Figure 2**. In terms of reference standards and flow and timing, all the included studies had a low risk of bias. However, there were several case–control comparative studies and the corresponding bias risk was high. The bias risk of 16 enrolled studies for index test was judged as high because the threshold was not prespecified. With regard to applicability concerns, the matching degree of all studies and evaluation questions were high.

### Meta−analysis

**Figure 3:**
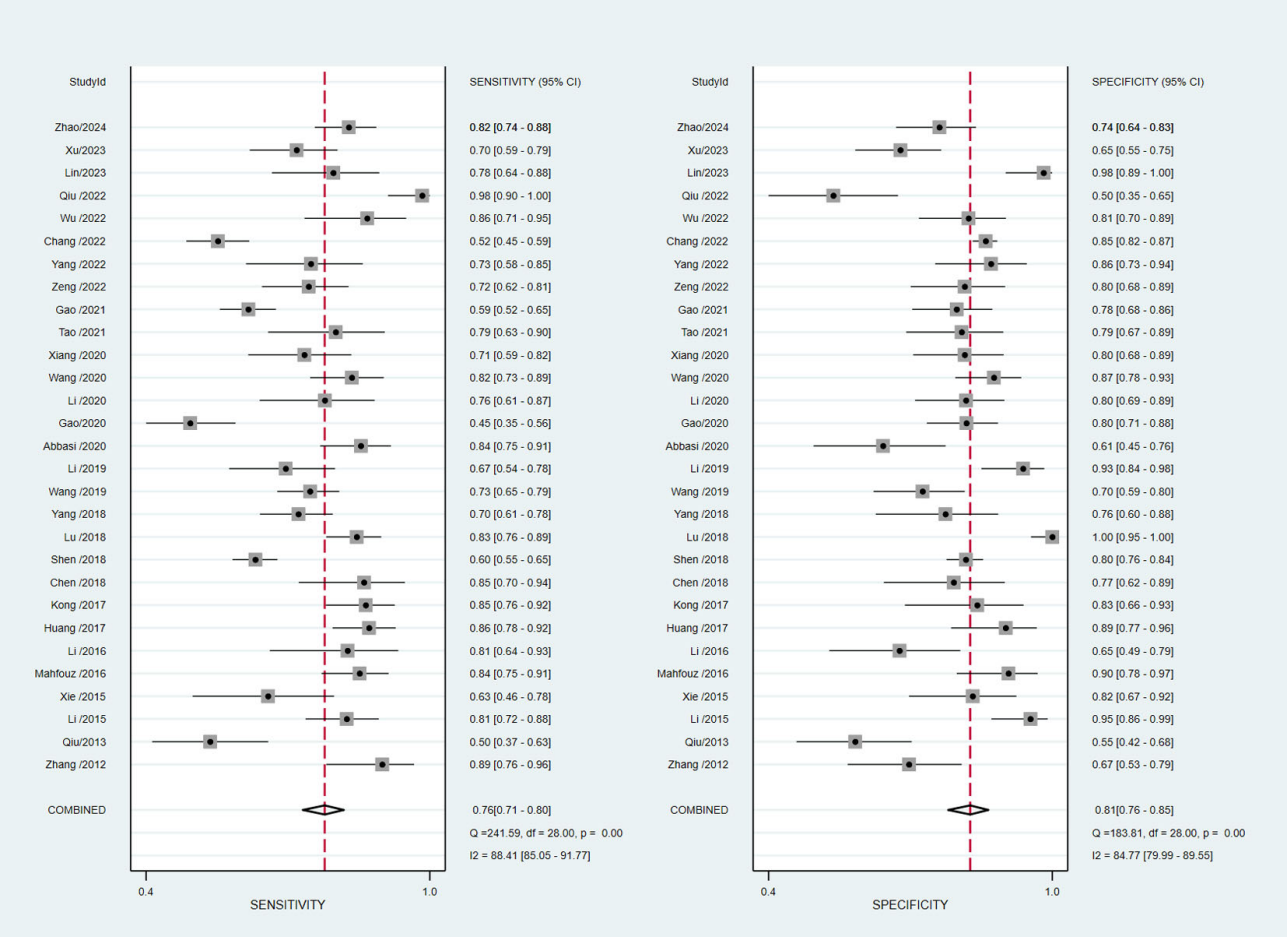
Forest plot of pooled sensitivity and specificity.

**Figure 4:**
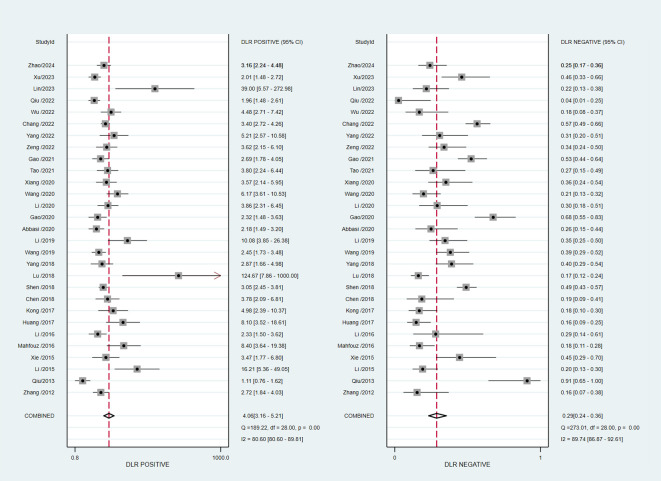
Forest plot of pooled positive likelihood ratio (PLR) and negative likelihood ratio (NLR).

**Figure 5:**
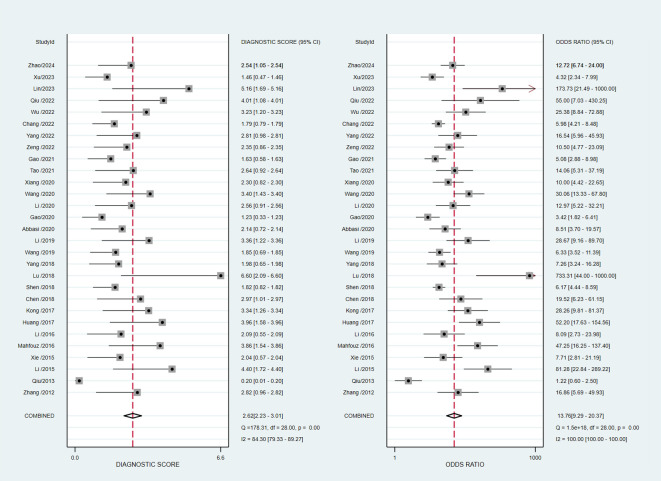
Forest plot of pooled diagnostic score and diagnostic odds ratio (DOR).

**Figure 6:**
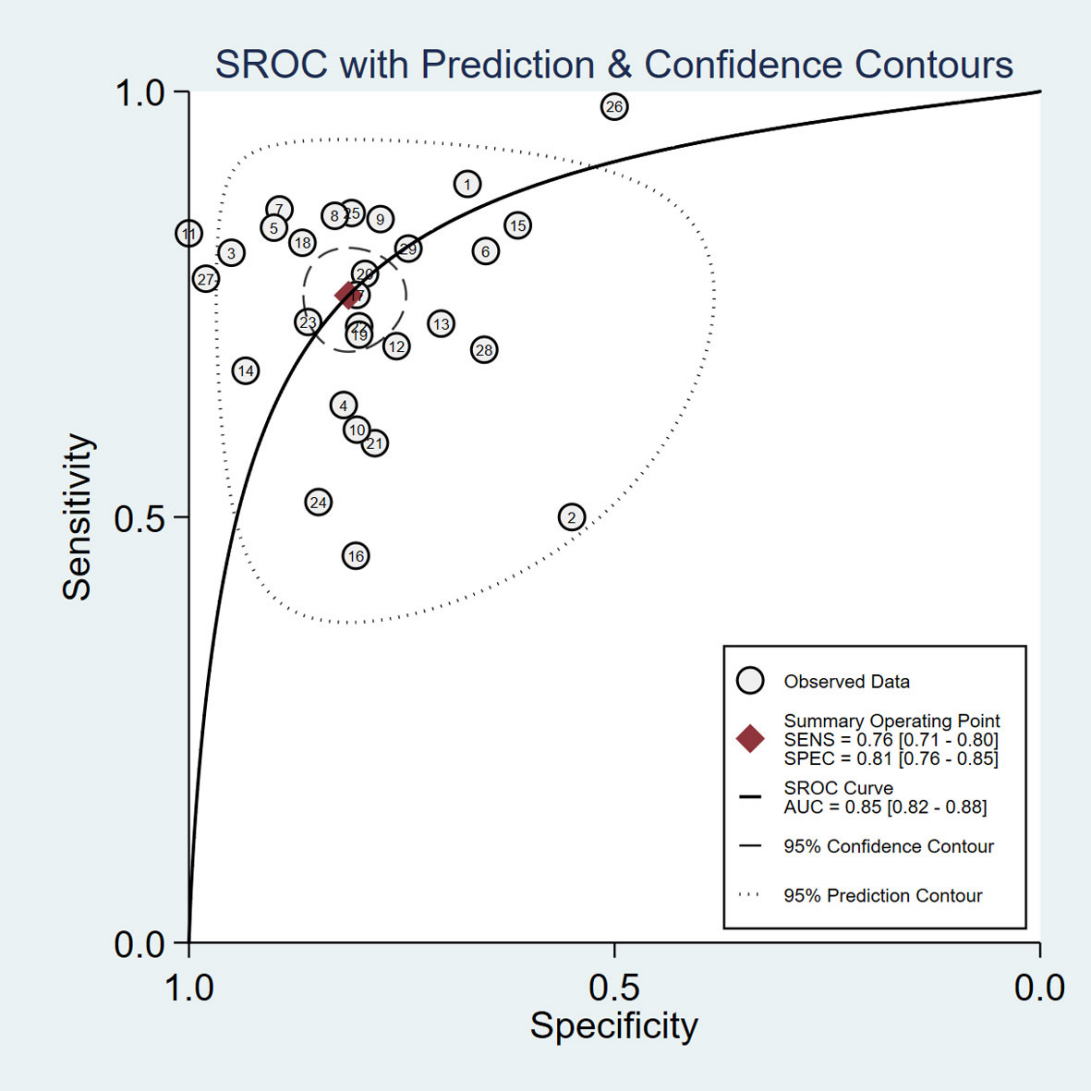
Summary receiver operating characteristic (SROC) plots.

**Figure 7:**
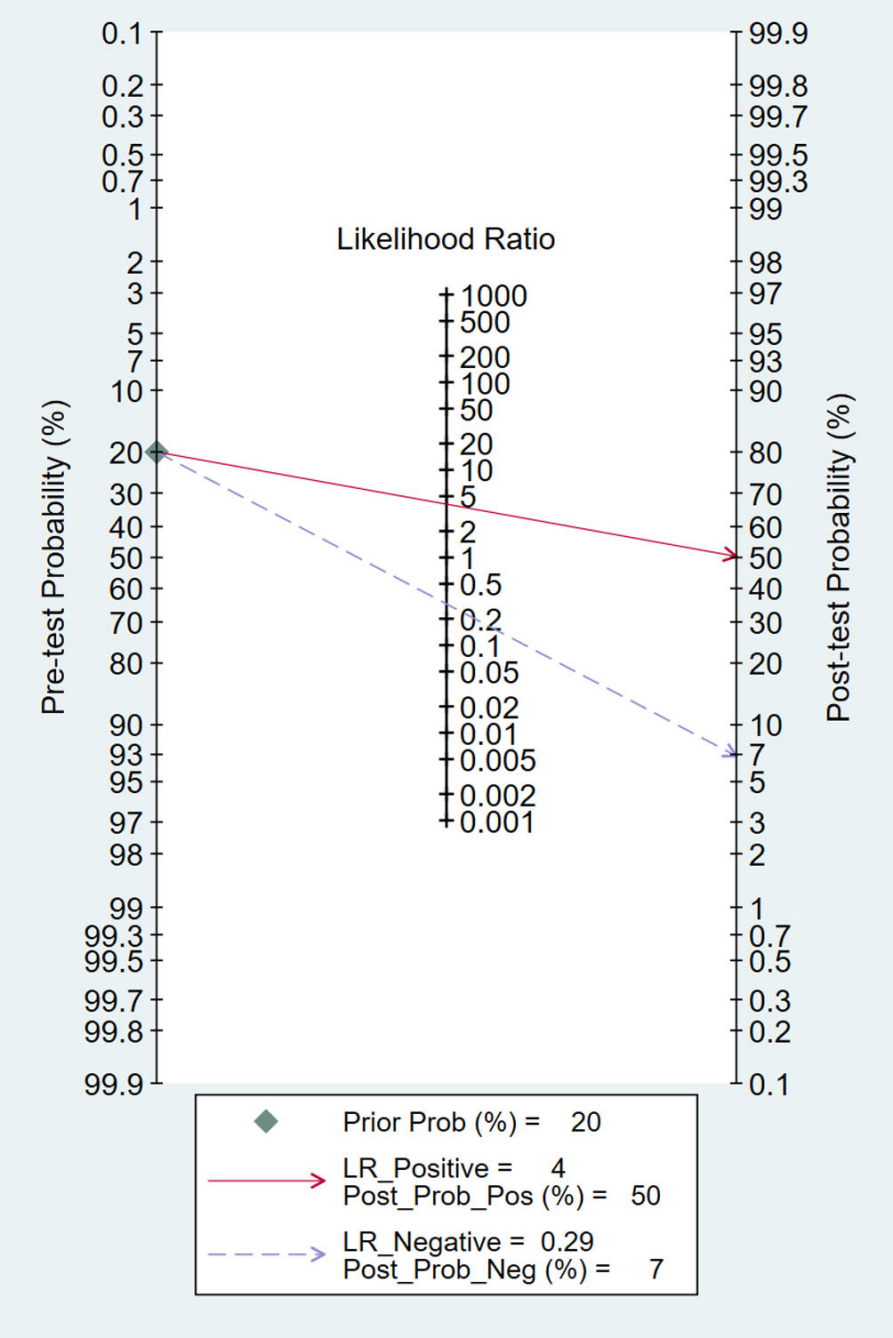
Fagan nomogram of retinol-binding protein 4 for the diagnosis of diabetic nephropathy.

The pooled diagnostic accuracy demonstrated the diagnostic value of RBP4 in T2DM with DN. The pooled sensitivity and specificity were 0.76 (95% CI, 0.71–0.80) and 0.81 (95% CI, 0.76–0.85), respectively (**Figure 3**). The heterogeneity was significant in the pooled analysis of sensitivity (*I* ^2 =^ 88.41, *P*<0.001) and specificity (*I^2 =^* 84.77, *P*<0.001). The pooled PLR was 4.06 (95% CI, 3.16–5.21) with significant heterogeneity (*I* ^2 =^ 80.60, *P*<0.001), and the pooled NLR was 0.29 (95% CI, 0.24–0.36) with significant heterogeneity (*I* ^2 =^ 89.74, *P*<0.001) (**Figure 4**). The pooled DOR was 13.76 (95% CI, 9.29–20.37), with significant heterogeneity (*I* ^2 =^ 100, *P*<0.001) (**Figure 5**). Additionally, the pooled summarized receiver operating characteristic (SROC) curve was calculated by sensitivity against (1 – specificity), and the AUC was 0.85 (95% CI, 0.82–0.88), revealing a high overall accuracy of RBP4 for T2DM with DN (**Figure 6**). The high diagnostic efficacy of RBP4 was confirmed by Fagan’s nomogram, with 50% and 7% for positive and negative post-test probability, respectively, when the pretest probability was set at 0.2 (**Figure 7**).

### Meta regression and sensitivity analyses

**Figure 8:**
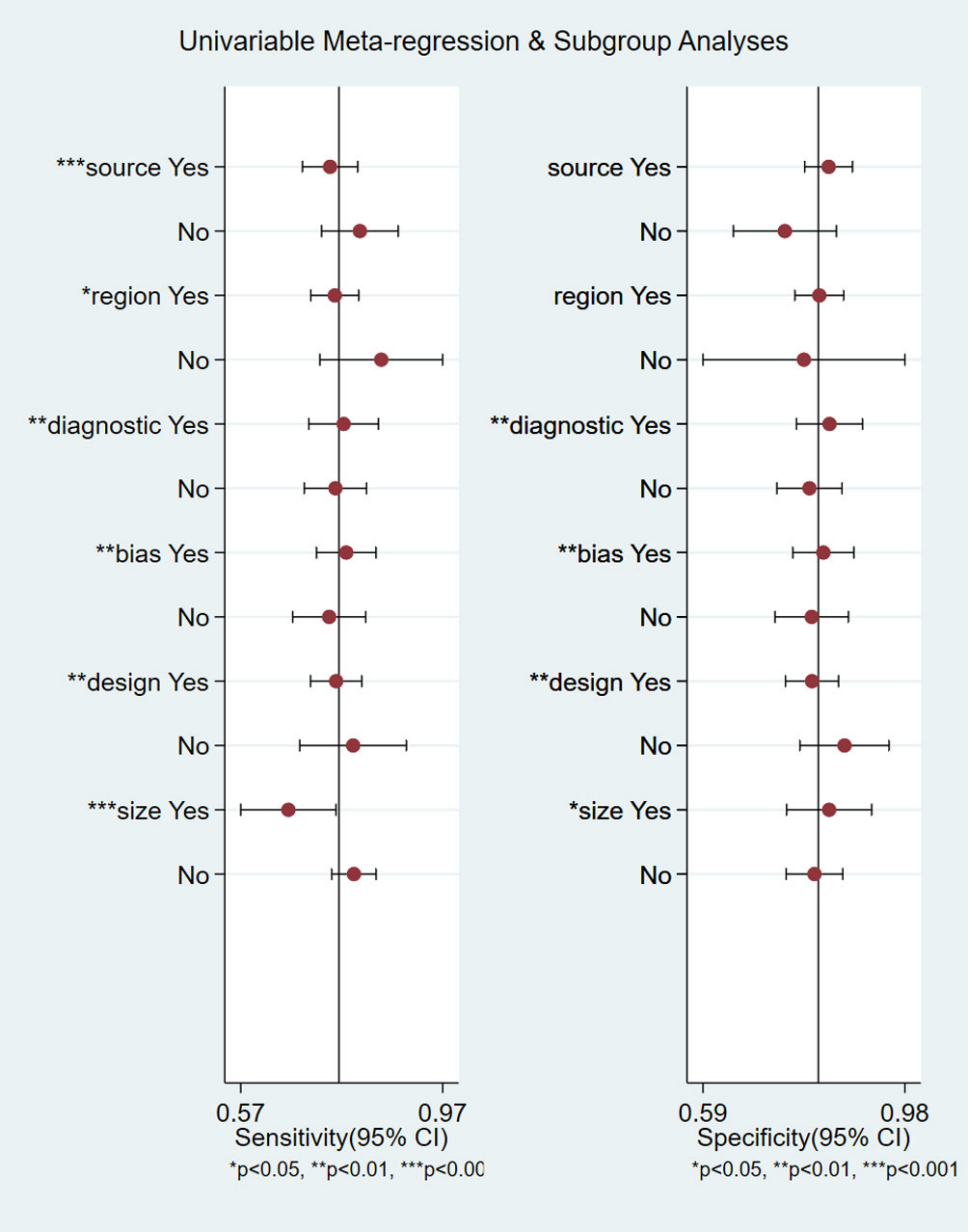
Meta-regression analysis for examining sensitivity and specificity of retinol-binding protein 4 for the diagnosis of diabetic nephropathy. *P<0.05, **P<0.01, ***P<0.001.

We performed meta-regression analysis with sample source, region, diagnostic criteria, bias risk for index test, study design and sample size as independent variables to explore the sources of heterogeneity (**Figure 8**). For sensitivity, six independent variables, sample source, region, diagnostic criteria, bias risk for index test, study design and sample size were statistically significant. For specificity, four independent variables, diagnostic criteria, bias risk for index test, study design and sample size were statistically significant. The results indicated that sample source, region, diagnostic criteria, bias risk for index test, study design and sample size were sources of heterogeneity.

**Figure 9:**
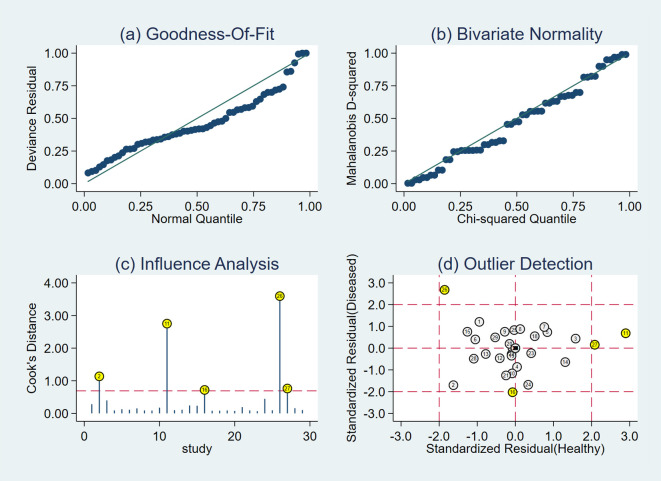
Diagram of sensitivity analysis showing **(A)** goodness-of-fit; **(B)** bivariate normality; **(C)** influence analysis; **(D)** outlier detection.

The results of the sensitivity analysis are shown in **Figure 9**. The goodness of fit (**Figure 9A**) and bivariate normality (**Figure 9B**) indicated that the random-effects model was applicable. Influence analysis showed that studies of Lu et al. (29) and Qiu et al. (39) were the most dominant studies in weight (**Figure 9C**). Outlier detection illustrated that heterogeneity might be attributed to the related data of Lu et al. (29) and Qiu et al. (39) (**Figure 9D**). After excluding the two outlier studies, the *I* ^2^ value of heterogeneity was reduced by 1.2% and 4.57% in sensitivity and specificity, respectively. There was no significant change in the pooled results for diagnostic efficacy (**Table 2**).

**Table 2:** Diagnostic performance of RBP4 in DN.

Analysis	Overall	Outliers excluded
No. of studies	29	27
Sen (95% CI)	0.76 (0.71–0.80)	0.74 (0.70–0.79)
Spe (95% CI)	0.81 (0.76–0.85)	0.81 (0.77–0.84)
PLR (95% CI)	4.10 (3.20–5.20)	3.80 (3.10–4.70)
NLR (95% CI)	0.29 (0.24–0.36)	0.32 (0.26–0.38)
DOR (95% CI)	14.0 (9.0–20.0)	12.0 (8.0–17.0)
AUC (95% CI)	0.85 (0.82–0.88)	0.85 (0.81–0.87)

### Publication bias

**Figure 10:**
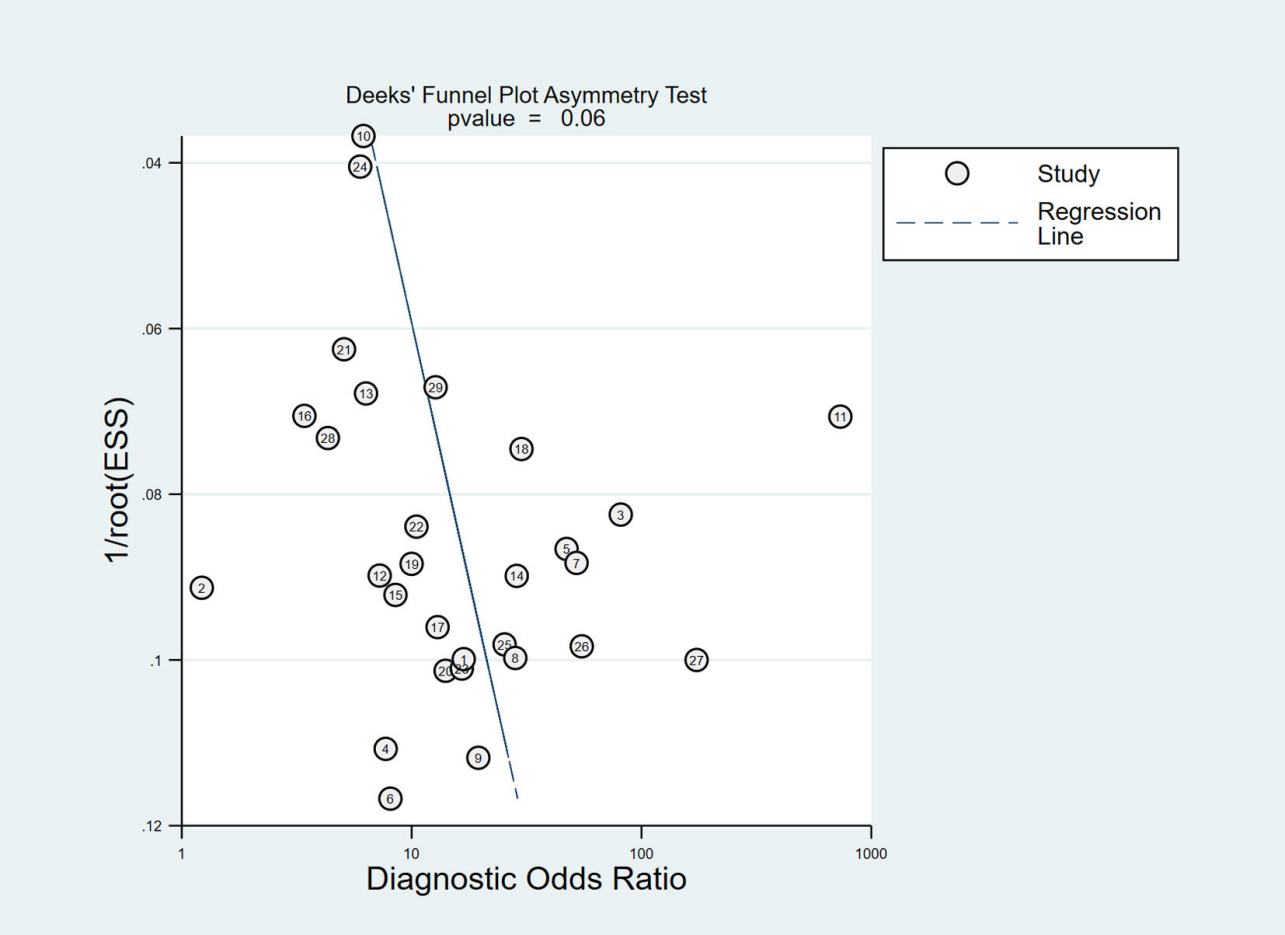
Deeks’ funnel plot asymmetry test for publication bias.

No obvious publication bias existed in the Deeks’ funnel plot asymmetry test (*P*=0.06) (**Figure 10**).

## Discussion

Early detection and prevention of DN, which is the major microvascular complication of DM and the main cause of end-stage renal disease (ESRD), are important (44). RBP4 has been considered as a single diagnostic marker for the detection of renal impairment (4, 14). Several studies have evaluated the relationship between RBP4 levels and early DN in patients with T2DM. Some studies have indicated an increase in serum RBP4 concentrations in T2DM patients with DN (4, 14, 15), and others have found similar RBP4 levels and a correlation with renal function and early DN in T2DM (16). Zhang et al. (45) conducted a meta-analysis to investigate the associations between RBP4 concentration and clinical indices of renal function and albuminuria in patients with T2DM. They demonstrated that RBP4 levels in the micro+macroalbuminuria group were significantly higher than those in the normal albuminuria group of patients with T2DM. The concentration of circulating RBP4 was positively correlated with ACR but negatively with eGFR. To our knowledge, a meta-analysis has not yet been conducted to explore the accuracy of the role of RBP-4 in diagnosis of DN in T2DM patients. Hence, we performed this study to evaluate the diagnostic value of RBP4 for early kidney damage in T2DM patients.

This meta-analysis included 29 original articles (5549 patients) with sufficient data for an investigation of the diagnostic accuracy of RBP4 in DN. The pooled sensitivity and specificity of RBP4 were 0.76 (95% CI, 0.71–0.80) and 0.81 (95% CI, 0.76–0.85), respectively. The likelihood ratio was useful for assessing the diagnostic value of the detection method. PLR>10 and NLR<0.1 demonstrated convincing diagnostic potential. The pooled PLR and NLR of RBP4 were 4.06 (95% CI, 3.16–5.21) and 0.29 (95% CI, 0.24–0.36), respectively, indicating that the diagnostic efficacy of RBP4 for DN was still limited. DOR, which combines sensitivity, specificity, PLR and NLR, is used as an independent indicator to determine diagnostic performance. The higher the DOR value, the better the discriminant effect of diagnostic indices. The pooled DOR in this meta-analysis was 13.76 (95% CI, 9.29–20.37), indicating good overall accuracy. The AUC of SROC for RBP4 was 0.85 (95% CI, 0.82–0.88), suggesting that RBP4 has a promising diagnostic accuracy for DN.

Xu et al. (46) investigated the association of serum RBP4 with impaired glucose regulation and microalbuminuria in Chinese adults aged ≥40 years. The results illustrated that serum RBP4 level was closely related with impaired glucose regulation and an independent risk factor for microalbuminuria. Chang et al. (47) indicated that serum RBP4 in patients with DM was positively associated with ACR and uric acid but negatively related with eGFR. Multiple stepwise linear regression analysis showed that uric acid and eGFR remained significantly correlated with serum RBP4. Mohamed et al. (14) performed a comparison between the output data of ROC curves for RBP4 and ACR to assess whether RBP4 was more sensitive and specific than ACR. A serum level of RBP4 >24.5 ng/mL predicted the presence of nephropathy with 84% sensitivity, 90% specificity, and AUC=0.912 with 86% accuracy; and urinary ACR >37.5 mg/g creatinine predicted the presence of nephropathy with 89% sensitivity, 72% specificity, and AUC=0.819 with 83.3% accuracy. These studies demonstrated a positive correlation between serum RBP4 and urine ACR and indicated that RBP4 was more specific than ACR for early prediction of DN.

The pathogenic mechanism explaining the differences in RBP4 levels in DM patients with and without renal dysfunction might be associated with reduced catabolism and IR. First, the kidneys play a critical role in maintenance of retinol homeostasis throughout the body, which is regulated by glomerular filtration and subsequent reabsorption of RBP4 into the proximal tubular tissues (48). Thus, disorder of renal function leads to accumulation of RBP4 in the plasma and hence to higher concentration in patients with DN than in T2DM patients without kidney disease (10). Second, RBP4 is a novel adipokine and increased circulating levels might be associated with deterioration of IR in patients with DN (6). This could result from increased expression of the gluconeogenic enzyme in live cells (mainly phosphoenolpyruvate carboxykinase), inhibition of insulin signaling, impairment of glucose uptake in skeletal muscle, resulting in higher glucose generation by the liver (49, 50).

Meta regression analyses suggested that the sample source, region, diagnostic criteria, bias risk for index test, study design and sample size might be the sources of heterogeneity. Higher sensitivity was found in the groups with urine samples, non-Chinese, ACR for detection of DN, bias risk for index test, case–control studies, and sample size ≤200 than in the corresponding groups. There was no significant difference in specificity between studies from serum and urine samples, China and other countries. Publication bias indicated that the findings were stable and reliable.

There were some limitations to the meta-analysis that need to be addressed when interpreting the results. Firstly, although we conducted an extensive literature search, there were no related studies from Europe or America. Secondly, information such as randomization and blindness were not stated in some studies. Thirdly, the heterogeneity in the present meta-analysis was obvious. In addition, some important factors, such as cut-off value and staging of DN were inconsistent among the studies. Therefore, investigation of the diagnostic value of RBP4 as a biomarker for early detection of DN needs a large sample, with blinding and randomization, using a unified detection method for DN staging, so that the authenticity and reliability of the analysis are more clinically meaningful.

In summary, this meta-analysis showed that RBP4 has promising diagnostic value with good sensitivity and specificity for patients with T2DM with DN. Considering the limitations of the present study, more high-quality research is needed to confirm the diagnostic potential of RBP4 in patients with DN.

## Data availability statement

The original contributions presented in the study are included in the article/**Supplementary Material**. Further inquiries can be directed to the corresponding author.

## Author contributions

XC: Writing – original draft, Software, Funding acquisition, Conceptualization. GZ: Writing – review & editing. TJ: Writing – original draft, Investigation. WH: Writing – review & editing, Investigation. JW: Writing – review & editing, Data curation. BS: Writing – review & editing, Methodology. RW: Writing – review & editing, Methodology.
